# Exploring the Barriers to Discussing Unconscious Racial Bias in Psychiatry Trainees

**DOI:** 10.1192/j.eurpsy.2022.2200

**Published:** 2022-09-01

**Authors:** D. Borges, T. Paris

**Affiliations:** Oxford Health NHS Foundation Trust, Warneford Hospital, Oxford, United Kingdom

**Keywords:** reflective practice, racial bias, institutional racism, medical education

## Abstract

**Introduction:**

Racism is present in most aspects of our society, including healthcare. Differences in health outcomes, and in the quality of mental health treatment for people coming from ethnic minority groups have been demonstrated in the literature. Psychiatry trainees are required to understand the impact of structural inequalities and power differences within mental health services, and to be able to deliver clinical care that is equitable for all.

**Objectives:**

To provide psychiatry trainees with a space to reflect on unconscious racial bias in clinical work and to explore potential barriers when talking about such topics.

**Methods:**

A Race and Equality Reflective Group for psychiatry trainees was organised as an opportunity to discuss unconscious racial bias. Due to an insufficient number of registrations, the session was cancelled. An anonymous feedback questionnaire was sent to all trainees to understand reasons behind this, and to explore potential barriers to participation. The results were analysed and were brought back to a regular Balint group for further exploration.

**Results:**

Twelve trainees filled in the questionnaire. The main themes identified included this topic being a sensitive issue (5; 41.7%), discomfort in trainees (5; 41.7%), insufficient time to participate (4; 33%) and timetable clash (3; 25.9%). Barriers to discussing unconscious racial bias and inequality were identified in further exploration with trainees. The tendency for groups to adopt a split position that was observed, mirrors the dynamics seen in institutional racism.

**Conclusions:**

This work has highlighted the need for ongoing focused, facilitated educational spaces where these issues can be openly discussed and reflected upon.

**Disclosure:**

No significant relationships.

